# Comparative Studies on Volatile Oil Components Derived from Integrated Processing Technology of Origin and Traditional Cutting Processing Technology of Moslae Herba and Their Effects on Rats With Lung-Yin Deficiency

**DOI:** 10.1155/2021/5557222

**Published:** 2021-05-25

**Authors:** Tian-Ni Jiang, Yue Liu, Hui Gao

**Affiliations:** College of Pharmacy, Liaoning University of Traditional Chinese Medicine, Shenyang, China

## Abstract

This study aimed to examine the components of the volatile oil derived from the integration processing technology of origin (IPTO) of Moslae Herba (MH) and their effects on the treatment of rats with Lung-Yin deficiency. IPTO was compared with the traditional cutting processing technology (TCPT) to provide a feasible basis. The chemical constituents of the volatile oil were identified by gas chromatography-mass spectrometry (GC-MS). The Lung-Yin deficiency model was established by subjecting the animals to smoke and administering them with thyroid tablet suspension. The rats were randomly divided into six groups: control, model, Tween-80, positive, IPTO, and TCPT. After 30 days of intragastric administration, the macroscopic signs of rats and pathological morphology of the lung tissues were observed by the hematoxylin and eosin (H & E) staining method. The positive expression of the tumor necrosis factor (TNF-*α*) was observed by an immunohistochemical method. The levels of cholesterol (CHO), albumin (ALB), total protein (TP), blood urea nitrogen (BUN), interleukin-1 (IL-1), interleukin-1beta (IL-1*β*), cyclic adenosine monophosphate (cAMP), and cyclic guanosine monophosphate (cGMP) were detected in the serum of rats. A total of 42 and 37 components were identified from the volatile oils of IPTO and TCPT, respectively. Among them, the contents of carvacrol and thymol were higher in IPTO. The volatile oil of MH derived from IPTO and TCPT could improve the general signs and autonomous activities of the rats with Lung-Yin deficiency; increase the contents of cGMP, TP, and ALB; and decrease the anal temperature of the rats, the contents of IL-1*β*, CHO, and BUN in serum, the ratio of cAMP to cGMP, and the average optical density of TNF-*α* in their lung tissues. The findings indicated that IPTO was more efficient than TCPT. Its therapeutic effect might be associated with the change in TNF-*α* expression; the increase in cGMP, TP, and ALB contents; and the decrease in IL-1*β*, CHO, and BUN levels, laying the foundation for the clinical development and application of MH.

## 1. Introduction

The processing of traditional Chinese medicine (known as “Pao Zhi” in Chinese) is a unique pharmaceutical technology. Crude drugs are processed using this technology based on the theory of traditional Chinese medicine, nature of the individual crude drug, and requirements of drug dispensing, pharmaceutical preparation, and clinical applications [1]. Following the initial simple processing of Chinese medicinal materials in the place of origin, additional processing is required for parts of Chinese medicinal decoction by cleaning, cutting, or certain heating methods, such as stir-baking, steaming, and calcination. These processes occur in the decoction pieces factory prior to the clinical use of the medicinal product. Inevitably, the processing occurs twice (once in the place of origin and subsequently in the decoction factory), and the medicinal components are easily lost. The State Administration of Traditional Chinese Medicine has initiated a project to study “the key technical specifications for integrating the origin and processing of traditional Chinese medicine.” The present study examined the traditional Chinese medicine Moslae Herba (MH). MH is the dried aerial part of *Mosla chinensis* Maxim or *Mosla chinensis* “Jiangxiangru.” The former is called “Qingxiangru,” and the latter is called “Jiangxiangru.” MH is also known as *Elsholtzia ciliata*, which is a famous traditional medicinal herb in China with an early historical record in “MING YI BIE LU” [2]. It is widely distributed in Jiangxi Province, China, and used as food and medicine. It thrives in summer and autumn [3]. MH is used for cold and fever, headache without sweating, vomiting and diarrhea, edema, and inhibition of urination [4]. The traditional cutting occurs initially followed by the production of the area cleaning, drying, and transport to the decoction pieces factory. Subsequently, water is added to soften, cut, and dry the material. It is a cumbersome process that can cause rancidity and mold formation and lead to the loss of effective ingredients. This preliminary study was conducted on the integration processing technology of origin (IPTO) of MH, and the preparation process of MH was established so as to simplify the tedious traditional cutting processing technology (TCPT) process of MH [5]; integration processing technology of origin (IPTO) includes washing, cutting without water softening, and drying, which can save resources more efficiently than TCPT. MH is used for both food and medicinal purposes. The volatile oil of MH exhibits antibacterial, antipyretic, analgesic, and antiviral activities [6]. In addition, it can cause intestinal propulsion and enhance immunity, which is considered the most important pharmacodynamic active ingredient in MH [7, 8]. In the present study, gas chromatography mass spectrometry (GC-MS) was used to qualitatively and quantitatively analyze the volatile oil content from IPTO and TCPT and examine the feasibility of IPTO of MH [9, 10]. The feasibility of the implementation of IPTO of MH was confirmed from the perspective of active components and content study. MH exerts antipyretic, anti-inflammatory, and therapeutic effects on rats with Lung-Yang deficiency [11, 12]. The traditional Chinese medicine theory states that the lungs are delicate and the nature tends to be soft and moist [13]. The lungs are considered as a purging organ; the Lung-Yin deficiency is the loss of nourishment or virtual fire, resulting in lung heat and loss of purging. The deficiency heat endophysis demonstrated this syndrome. Lung heat is the basis of Lung-Yin deficiency, and heat is the manifestation of inflammatory reaction. Hence, the levels of inflammatory factors, TNF-*α* and IL-1, increases significantly. The main clinical symptoms of Lung-Yin deficiency are cough without phlegm, dry mouth and dry throat, body loss, and other common symptoms. In the present study, the effects of the volatile oil of IPTO and TCPT were assessed on rats with Lung-Yin deficiency. The associated mechanism of action was also explored. The Baihe Gujin tablet is composed of 10 traditional Chinese medicines, including *Angelicae sinensis* Radix, Scrophulariae Radix, Paeoniae Radix Alba, and Glycyrrhizae Radix et Rhizom. It has the effects of nourishing Yin, moistening the lung, resolving phlegm, and relieving cough, Therefore, it is used for the deficiency of Yin in the lung and kidney, dry cough with less phlegm, and sore throat. In the present study, the Lung-Yin deficiency model was established by subjecting the animals to smoke and administering them with thyroid tablet suspension. The principle was to use thyroxine to cause Yin deficiency first and then subject them to smoke to cause cough. If the cough is not cured for a long time, it damages Lung-Yin [14]. The Baihe Gujin tablet is then used as a positive drug in the pharmacological experiment to compare the volatile oil components derived from IPTO and TCPT of MH and their effects on the treatment of rats with Lung-Yin deficiency. The pharmacodynamic study focused on the feasibility of IPTO, laying the foundation for the clinical development and application of MH.

## 2. Materials and Methods

### 2.1. Material

#### 2.1.1. Equipment Used in This Study

The following equipment was used in this study: TRACE1310-ISQ Gas Chromatography-Mass Spectrometer (Thermo Fisher Scientific, USA); NIST2.2 database (Thermo Fisher Scientific, USA); Multiskan MK3 Microplate reader (Thermo Fisher Scientific, USA); electrothermal constant temperature water bath pot (Changzhou Xiangtian Laboratory Instrument Factory, China); HZ-A6002 electronic balance (Ruian Jinxun Trading Co., Ltd., China); 2235-Leica paraffin embedding machine (Shanghai Qiwei Industrial Co., Ltd., China); Animal-6008 blood analyzer (Jinan Gelite Technology Co., Ltd., China); SHA-6 water bath constant temperature oscillator (Changzhou Guohua Electric Appliance Co., Ltd., China); Olympus BS-53 microscope (Zhonghui Laibo Instrument Co., Ltd., China); H1650-W Xiangyi centrifuge (Hunan Xiangyi Test Instrument Development Co., Ltd.,China); and Loyola Induction cooker (Guangdong Shunde Zhongchen Electric Co., Ltd.,China).

#### 2.1.2. Reagents Used in This Study

The following reagents were used in this study. MH was collected by our research group in Xinyu county, JiangXi Province, China. It was identified as the dry part of *Mosla chinensis* Maxim, a Labiaceae plant, by professor Zhai-yanjun of Liaoning University of Traditional Chinese Medicine. Baihe Gujin tablets (Guangdong Wanfang Pharmaceutical Co., Ltd., National Pharmaceutical Standard Z20090800); cholesterol (CHO) (lot number 2016011); albumin (ALB) (lot number 2016022), total protein (TP) (lot number 2016022); urea (BUN) (lot number 2016005) (Changchun Huili Biotechnology Co., Ltd.,China); interleukin-1 (IL-1) (lot number BPE30418); interleukin-1beta (IL-1*β*) (lot number BPE30419); cyclic adenosine monophosphate (cAMP) (lot number BPE30574); cyclic guanosine monophosphate (cGMP) (lot number BPE30335) (Shanghai Langdon Biotechnology Co., Ltd.,China); and Red plum filter cigarette (tar amount: 10 mg, flue gas nicotine amount: 0.8 mg, flue gas carbon monoxide amount: 12 mg; Hongta Tobacco Co., Ltd.,China) were used. Ether, formaldehyde, and other reagents were of analytically pure grade.

#### 2.1.3. Experimental Animals

Sixty specific pathogen-free (SPF) Sprague Dawley (SD) male rats, with a mean weight of 170 ± 10 g, were purchased from the Liaoning Changsheng Biotechnology Co., Ltd. (Animal License NO. SCXX 2017-0001). All experiments involving animals were approved by the Animal Core and Welfare Committee of Liaoning University of Traditional Chinese Medicine (use license number: SYXK (Liao) 2019-0004).

### 2.2. Methods

#### 2.2.1. Extraction of Volatile Oil of the IPTO and TCPT by GC-MS

IPTO decoction pieces: after the clean preparation of MH, the raw material was cut without softening into 1.0 cm and dried at 50∼60°C for 36 hours [5].

TCPT decoction pieces: after the clean preparation of MH, they were dried and softened with water for 1 hour, cut into 1.0 cm, and dried at 50–60°C for 36h.

Volatile oil extraction: 60 g decoction pieces of MH were accurately weighed, placed in the volatile oil extractant, extracted by steam distillation for 5 hours, extracted with ethyl ether, and dried with anhydrous sodium sulfate, ethyl ether was recovered, and the light yellow volatile oil with special strong fragrance is obtained and reserved. The extraction rate of volatile oil from MH was 1.06% (ITPO) and 0.94% (TCPT), respectively.

#### 2.2.2. The IPTO and TCPT on the Volatile Oil of MH by GC-MS Analysis

Chromatographic column: HP-5MS quartz capillary column (30 m × 0.25  mm × 0.25 *μ*m). Column temperature: the initial temperature was 60°C, keeping for 2 minutes, increasing to 190°C at 5°C/min, keeping for 6 minutes, and then, increasing to 250°C at 5°C/min, keeping for 5 minutes. The carrier gas was helium. The split ratio was 50 : 1. The inlet temperature was 250°C. The column flow rate was 110°C/min. The ion source was EI source, the electron bombardment energy was 70 EV, the doubling voltage was 1.765 kV, the ion source temperature was 230°C, and the scanning range was 35–550 amu. Each volatile oil sample was diluted with ether for one time, then injected and detected by GC-MS. Mass spectrogram was obtained by scanning the peaks in the total ion flow diagram. After retrieving the NIST 2.2 mass spectrogram database and combining with the manual spectrum analysis, the mass spectrogram of the peaks was checked with the literature to determine the chemical components in the volatile oil of MH. The relative percentage content of each component was determined by the peak area normalization method.

#### 2.2.3. Drug Preparation

The volatile oil was extracted according to the method of determination of volatile oil in Chinese Pharmacopoeia [1]. The extracted volatile oil was prepared from distilled water containing 2% Tween-80 (20 mL) into two volatile oil solutions of 1000 mL, and the amount of crude drug was 0.6252 g·mL^−1^ [15]. The positive control solution was prepared by dissolving Baihe Gujin tablets with distilled water. The dosage was 0.3125 mL·kg ^−1^·d^−1^ [15].

#### 2.2.4. The Lung-Yin Deficiency Model Rats and Treatment

After 1 week of adaptive feeding, the rats were randomly divided into six groups: a normal control group, model group, Tween-80 group, positive drug group, IPTO volatile oil group, and TCPT volatile oil group, with 10 rats in each group. Except for the normal control group, the other groups were subjected to smoke in a self-made fumigation box for 30 min every day (cigarette: 3 cigarettes/box) and administered with intragastric thyroid tablet solution (1 mL/100 g) for 30 days. The general signs and spontaneous activity of the rats were observed. After successful modeling, each group was given thyroid tablet solution (1 mL/100 g) for 30 days intragastrically. The rats in the normal control and model groups were given distilled water, the rats in the Tween-80 group were given 2% Tween-80, and the rats in the positive drug group were given Lilium solid gold tablet solution intragastrically. The volatile oil of MH was given to rats in each administration group. After 60 days, blood was collected from the abdominal aorta. The heart, liver, spleen, lung, and kidney were removed from each rat and weighed.

#### 2.2.5. Observation of Macroscopic Index

In the course of the experiment, the autonomic activity and general physical signs of the rats were observed closely every day, and the body weight, eating and drinking, and the change of anal temperature of the rats were recorded at the beginning of the experiment, after the establishment of the model, and after the treatment.

#### 2.2.6. Measurement of Serum Biochemical Indicators

The blood samples were collected from the abdominal aortic for 5 mL and placed in an EP tube without anticoagulant, standing at 25°C for 1 hour, centrifuged for 10 min at 8000 rpm. Serum was used to measure the levels of CHO, ALB, TP, BUN, IL-1, IL-1*β*, cAMP, and cGMP. The operation was carried out according to the instructions in the kit.

#### 2.2.7. Pathological Observation and Immunohistochemistry of the Lung

The part of the right lobe of the liver was fixed in 10% formalin from each rat. Tissues were processed and embedded in paraffin wax, and tissue sections were cut onto glass slides. Sections of the rat lung were routinely stained with hematoxylin and eosin for light microscopy. The immunopositive expression of the tumor necrosis factor-*α* (TNF-*α*) in lung tissue was detected by SABC. Immune expression image analysis: the positive expression of TNF-*α* was found in the cytoplasm of cells with clear brown and yellow granules under a light microscope. The expression results of immunohistochemistry were analyzed by using a multifunctional image analysis system, and the total positive expression area and average optical density were quantitatively analyzed.

#### 2.2.8. Organ Coefficient

After the animals were killed, the heart, liver, spleen, lungs, and kidney were taken out, weighed on the electronic balance immediately, and the organ coefficient was calculated: organ coefficient = organ mass/body weight × 100%.

## 3. Statistical Analysis

The measurement data were expressed as the mean ± standard deviation. Data were analyzed using SPSS version 23.0 software. After statistical analysis of the data of each group by ANOVA analysis of variance, the *t* test was used for comparison between the groups [16]. The test level *a* was 0.05 or 0.01. A *P* value˂0.05 or 0.01 was considered to be statically significant.

## 4. Results and Discussion

### 4.1. Results

#### 4.1.1. GC-MS Analysis of the Volatile Oil of IPTO and TCPT

**Figure 1:**
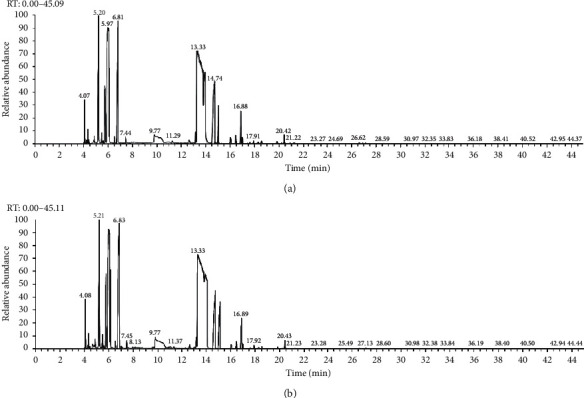
Total ion chromatogram (TIC) of the IPTO group (a) and TCPT group (b) by GC-MS.

The volatile oil of IPTO and TCPT of MH was detected by GC-MS. The total ion chromatogram is shown in Figure 1. 42 components were identified in the volatile oil of IPTO and 37 components in TCPT, and the chemical structure of these components is shown in Table 1. The carvacrol and thymol are the main index components in MH, and their contents are higher in the IPTO samples.

#### 4.1.2. Observation of Macroscopic Index

After half of the month, in the model, the rats appeared to have low body weight, a decrease in food consumption, and a significant increase in the anal temperature, and the model was successful (Table 2). After the treatment of the volatile oil, the changes of the anal temperature in the first 16 days were not obvious, and the anal temperature of the IPTO volatile oil group on the 25th day was significantly lower than that in the model group (*P* < 0.05).

#### 4.1.3. Serum Index Level

**Figure 2:**
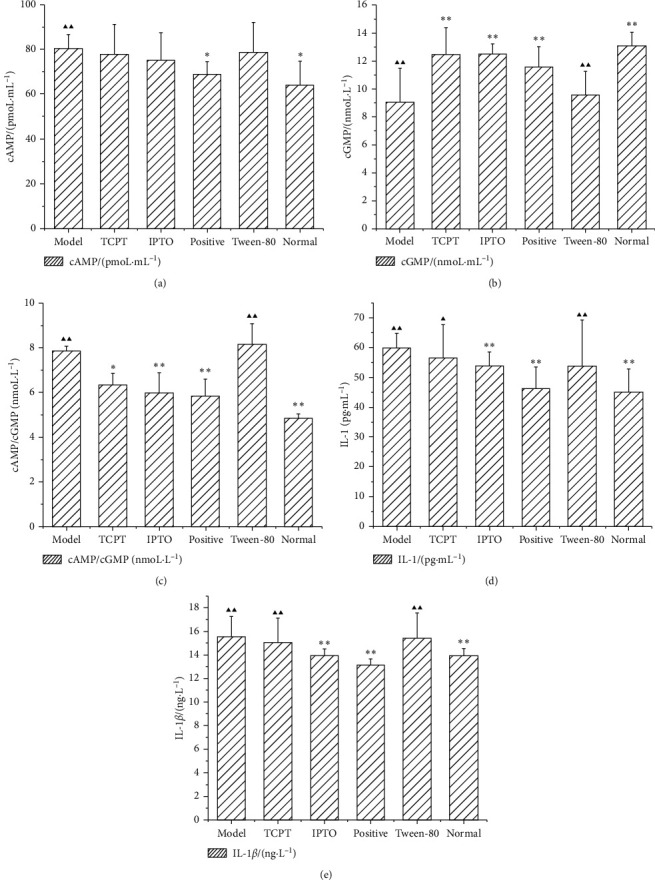
Effects of the IPTO and TCPT of MH on cAMP (a), cGMP (b), cAMP/cGMP (c), IL-1 (d), and IL-1*β* (e) levels of rats with deficiency of Lung-Yin ($\bar{X}$ ± *s*, *n* = 10). ^▲^*P* < 0.05, ^▲▲^*P* < 0.01 vs. normal group, ^*∗*^*P* < 0.05, ^*∗∗*^*P* < 0.01 vs. model group.

Compared with the normal group, the serum levels of IL-1, IL-1*β*, and cAMP and the ratio of cAMP to cGMP in the model group rats were increased, the cGMP was decreased, and the differences were statistically significant (*P* < 0.05,  0.01) (Table 3). The serum cGMP content of the TCPT and IPTO groups was significantly increased compared with that of the model group (*P* < 0.01) (Figures 2(a) and 2(b), and the difference was statistically significant (*P* < 0.01). The serum ratio of cAMP to cGMP in the TCPT and IPTO groups was decreased compared with that of the model group, and the differences were statistically significant (*P* < 0.05,  0.01) (Figure 2(c)). Current studies have suggested that cAMP is similar to the Yang of Chinese medicine and that cGMP is similar to the Yin of Chinese medicine. The effects caused by cAMP and cGMP are the opposite. For patients with Yin deficiency and fire, the ratio of cAMP to cGMP is significantly higher than normal [17].

The IPTO and TCPT groups caused an apparent increase in the cGMP content of the rats, whereas they did not exhibit a significant effect on the cAMP content, and the ratio of cAMP to cGMP decreased. The IPTO group exhibited a reduction in the content of IL-1*β* in the serum, whereas the TCPT group did not. The effects noted on IL-1 were not significant for both groups (Figures 2(d) and 2(e)). Compared with the normal group, the serum levels of CHO and BUN in the model group were increased, while the contents of TP and ALB decreased (Table 4). The differences were statistically significant (*P* < 0.05,  0.01). Compared with the model group, the levels of TP and ALB in the serum of the IPTO group were increased, and CHO and BUN contents were decreased; the difference was statistically significant (*P* < 0.05,  0.01). But, only the BUN difference in the TCPT group was statistically significant (*P* < 0.01).

#### 4.1.4. Pathological Observation and the Immunohistochemical Method of the Lung

**Figure 3:**
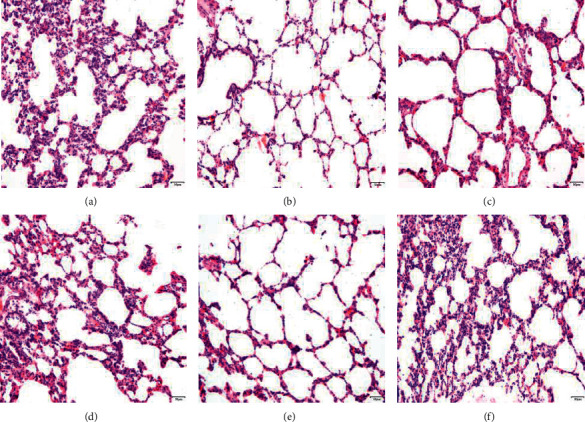
Pathological characteristics of lung tissue in rats of the model group (a), positive group (b), normal group (c), Tween-80 group (d), IPTO group (e), and TCPT group (f) (HE × 200, *n* = 10).

**Figure 4:**
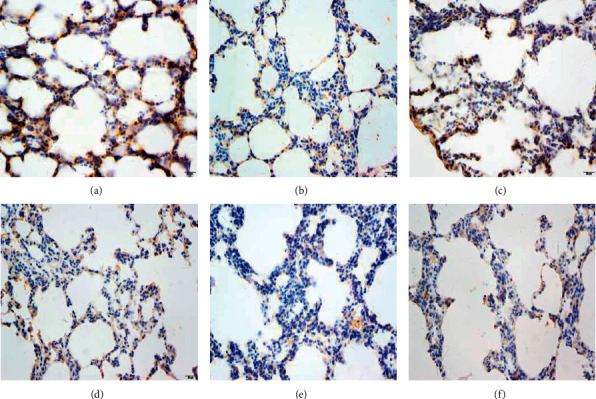
Immune expression image analysis of lung tissue in rats of the model group (a), positive group (b), normal group (c), Tween-80 group (d), IPTO group (e), and TCPT group (f).

In the normal group, the morphology and structure of the lung were normal, the alveolar wall was not significantly thickened, the alveolar septum structure was clear, there was no inflammatory cell infiltration in the pulmonary stroma, and the alveolar size was uniform (Figure 3). In the model group, the alveolar wall was congestive and had edema, part of the alveolar cavity was enlarged, the alveolar wall became thinner, broken, or converged, there was a large number of inflammatory cell infiltration, epithelial cell degeneration, necrosis, or exudation, and exudates could be seen in the alveolar stroma. Exudate was still found in the alveolar stroma of the Tween-80 group similar to the model group. Both the MH group and positive drug group improved the pathological structure of the lung, recovered the alveolar cavity, decreased or disappeared neutrophils, monocytes, and eosinophils, and no epithelial cell degeneration, necrosis, or exfoliation were found, and the ITPO group and positive drug group had a significant effect. Compared with the normal group, the abnormal index and the average optical density of TNF-*α* in the lung tissue of the model group of deficiency of Lung-Yin increased significantly (*P* < 0.05,  0.01) (Figure 4). Compared with the model group, the abnormal index of TNF-*α* in the volatile oil of MH (IPTO and TCPT) has a significant difference (*P* < 0.05,  0.01) (Table 5).

#### 4.1.5. Organ Coefficient

Compared with the normal group, the heart, liver, spleen, lung, and renal organ coefficients of the model group were increased (Table 6), and the differences were statistically significant (*P* < 0.05,  0.01). Compared with the model group, the heart and lung coefficients of the IPTO group were all reduced, and the difference was statistically significant (*P* < 0.05).

### 4.2. Discussion

In the present study, MH was processed by two different methodologies, respectively, which are integration processing technology of origin and traditional cutting processing technology. The yields of the volatile oil from the two processing methods were 1.06% (IPTO) and 0.94% (TCPT), respectively. The IPTO volatile oil content was significantly higher than that of the TCPT. The results indicated that IPTO could avoid the destruction and loss of active components. GC-MS analysis demonstrated that 67 compounds were detected, of which 42 components were identified by IPTO and 37 by TCPT. Trans-caryophyllene [18], *β*-sesquiphellandrene [19], and *α*-terpineol [20, 21] can be used for repairing and protecting the cells. They also exhibit an antibacterial effect. Caryophyllin possesses an anti-inflammatory effect [20].The historical pharmacopoeia states that the active ingredients of MH are thymol and carvacrol. Both of these components were found at high contents in the IPTO. Concomitantly, a previous study has shown no significant difference in the single antibacterial ability between carvacrol and thymol. A 2 : 1 ratio of thymol to carvacrol exhibited the highest antibacterial effect, whereas the synergistic effect was better than that noted from the same dose of thymol and carvacrol [22].The ratio of thymol and carvacrol in the IPTO group was approximately 2 : 1. Therefore, the IPTO could retain the active components more efficiently than TCPT.

MH is a traditional Chinese medicine acting on the lung and stomach meridian. According to the cause and pathogenesis of the statement “The heat is arrogant, and the lung-fluid is burned” of the traditional Chinese medicine theory, the Lung-Yin deficiency animal model is prepared by smoking and by administrating a thyroid tablet [23]. The expression levels of IL-1 and IL-1*β* were increased, and the regulation and control were the essence of the Lung-Yin deficiency syndrome [24]. At present, it is considered that cAMP and cGMP are similar to the Yang and Yin of traditional Chinese medicine and that their effects are the opposite [15]. Therefore, the present study examined the levels of IL-1, IL-1*β*, cAMP, and cGMP in the serum of rats with Lung-Yin deficiency. In the present study, MH exhibited a therapeutic effect in rats with deficiency of Lung-Yin by increasing cGMP, TP, and ALB content and by decreasing IL-1*β*, CHO, and BUN content. MH regulated substance metabolism and inhibited TNF-*α* expression, which was not associated with cAMP and IL-1 levels. The effects of the volatile oil of TCPT were not apparent. In particular, the improvement effect of the volatile oil derived from IPTO was optimal.

## 5. Conclusions

The chemical components of the essential oil were identified by GC-MS, and the relative contents of each component were calculated. The yield of volatile oil from IPTO was significantly higher than that from TCPT. Therefore, the destruction and loss of active components could be avoided using IPTO. The results of pharmacological experiments showed that the therapeutic effect of volatile oil extracted from IPTO was better than that of TCPT in treating rats with Lung-Yin deficiency by MH. The therapeutic effect might be related to the change in TNF-*α* expression; the increase in cGMP, total protein (TP), and albumin (ALB) contents; and the decrease in IL-1*β*, cholesterol (CHO), and BUN levels. The present study assessed the contents of the active components of MH and their biological and pharmacodynamic effects on rats with Lung-Yin deficiency. The data provided a theoretical basis for the implementation of the integration processing technology of MH and a reference for the processing, development, and application of MH components.

## Figures and Tables

**Table 1:** The results of GC-MS analysis on chemical composition and the structure of the volatile oil from MH of IPTO and TCPT.

No.	Compounds	Molecule	Structure	Mr	Relative contents (%)	
					IPTO	TCPT
1	Pinene	C_10_H_16_	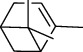	136	1.47	1.66
2	Bicyclo [3.1.0]hex-2-ene, 4-methylene-1-(1-methylethyl)-	C_10_H_14_	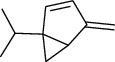	134	0.29	0.20
3	Camphene	C_10_H_16_	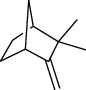	136	0.53	0.55
4	Benzaldehyde	C_7_H_6_O	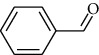	106	0.09	0.40
5	Sabinene	C_10_H_16_	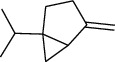	136	0.13	0.40
6	*β*-Pinene	C_10_H_16_	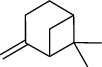	136	0.31	–—
7	Myrcene	C_10_H_16_	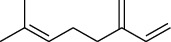	136	7.20	7.18
8	*α*-Phellandrene	C_10_H_16_	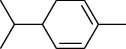	136	0.56	–—
9	3-Carene	C_10_H_16_	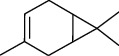	136	0.13	0.13
10	4-Isopropyltoluene	C_10_H_14_	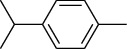	134	21.84	6.01
11	1, 8-Cineole	C_10_H_18_O	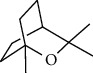	154	2.60	21.11
12	Ocimene	C_10_H_16_	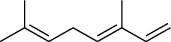	136	0.34	2.72
13	*γ*-Terpinene	C_10_H_16_	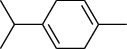	136	17.09	18.90
14	Cyclohexanol, 1-methyl-4-(1-methylethenyl)-, cis-	C_10_H_18_O	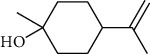	154	0.05	0.15
15	Terpinolene	C_10_H_16_	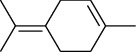	136	0.30	0.35
16	1-Methyl-4- (1-methylvinyl) benzene	C_10_H_12_	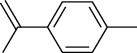	132	0.08	0.05
17	Linalool	C_10_H_18_O	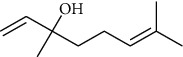	154	0.07	0.08
18	Terpinen-4-ol	C_10_H_18_O	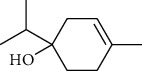	154	0.98	1.20
19	2-(4-Methylphenyl) propylene-2-alcohol	C_10_H_14_O	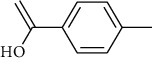	150	0.03	–—
20	*α*-Terpineol	C_10_H_18_O	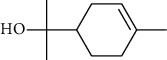	154	0.12	0.10
21	Bicyclo [3.1.0]hex-3-en-2-one, 4-methyl-1-(1-methylethyl)-	C_10_H_14_O	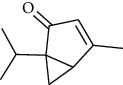	150	0.18	0.14
22	(Z)-carveol, 2-methyl-5-(1-methylethenyl)-2-cyclohexen-1-ol,cis-mentha-1, 8-dien-6-ol	C_10_H_16_O	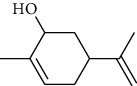	152	0.04	–—
23	(+)-2-Carene	C_10_H_16_	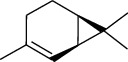	136	0.38	–—
24	Verbenol	C_10_H_14_O	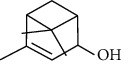	150	0.04	–—
25	(−)-trans-Myrtanol	C_10_H_18_O	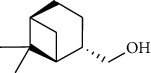	154	0.07	–—
26	Geraniol	C_10_H_18_O	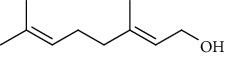	154	0.79	0.67
27	Thymol	C_10_H_14_O	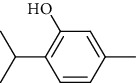	150	25.88	13.44
28	5-Methyl-2-(1-methylethyl)-	C_12_H_16_O_2_	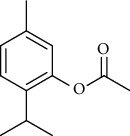	192	10.82	8.35
29	Eugenol	C_10_H_12_O_2_	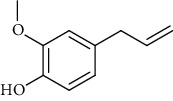	164	0.07	–—
30	Carvacryl acetate	C_12_H_16_O_2_	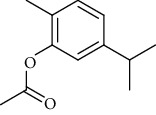	192	2.69	4.92
31	trans-Caryophyllene	C_15_H_24_	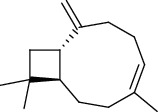	204	0.47	0.29
32	2, 6-Dimethyl-6-(4-methyl-3-pentenyl)bicy-clo [3.1.1] hept-2-ene	C_15_H_24_	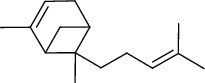	204	0.55	0.45
33	*α*-Caryophyllene	C_15_H_24_	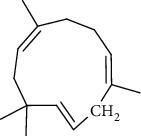	204	2.18	2.02
34	(E)-7, 11-dimethyl-3-methylene-1, 6, 10-dod-ecanetriene	C_15_H_24_	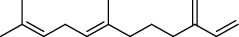	204	0.33	0.27
35	(E)-*β*-farnesene	C_15_H_24_	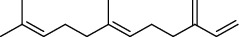	204	0.09	0.06
36	(Z, E)-*α*-farnesene	C_15_H_24_	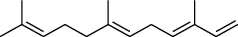	204	0.18	0.17
37	*ç*-Mullorene	C_15_H_24_	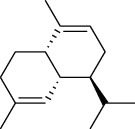	204	0.13	–—
38	*β*-Sesquiphellandrene	C_15_H_24_	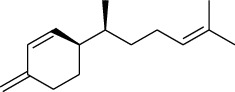	204	0.20	0.16
39	Caryophyllin	C_15_H_24_O	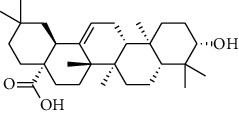	220	0.17	0.14
40	Humulene epoxide II	C_15_H_24_O	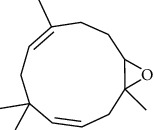	220	0.46	0.42
41	T-cadinol	C_15_H_26_O	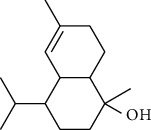	222	0.06	0.04
42	Carvacrol	C_10_H_14_O	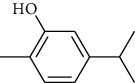	150	10.78	8.41
43	Mushroom alcohol	C_8_H_16_O	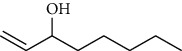	128	–—	0.07
44	*α*-Thujone	C_10_H_16_O	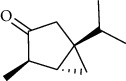	152	–—	0.06
45	DL-isoborneol	C_10_H_18_O	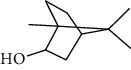	154	–—	0.05
46	L (−)-carvone	C_10_H_14_O	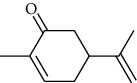	150	–—	0.14
47	(1*α*, 2*β*, 5*α*)-2-Methyl-5-(1-methylethyl) bicyclo [3.1.0] hexan-2-ol	C_10_H_18_O	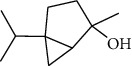	154	–—	–—
48	trans-1-Methyl-4-(1-methylvinyl)cyclohex-2-en-1-ol	C_10_H_16_O	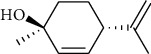	152	–—	–—
49	Borneol	C_10_H_18_O	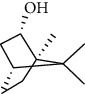	154	–—	–—
50	(−)-Terpinen-4-ol	C_10_H_18_O	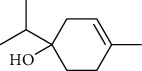	154	–—	–—
51	Bornyl formate	C_11_H_18_O_2_	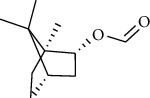	182	–—	–—
52	Citronellol	C_10_H_20_O	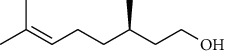	156	–—	–—
53	Ascaridole	C_10_H_16_O_2_	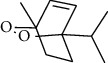	168	–—	–—
54	Germacrene D, 1-methyl-5-methylene-8-(1-methylethyl)-1, 6-cyclodecadiene	C_15_H_24_	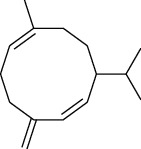	204	–—	–—
55	Zingiberene	C_15_H_24_	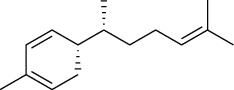	204	–—	–—
56	*α*-Muurolene	C_15_H_24_	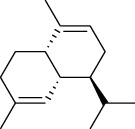	204	–—	–—
57	1-Isopropyl-7-methyl-4-methylene-1, 2, 3, 4, 4a, 5, 6, 8a-octahydro-naphthalene	C_15_H_24_	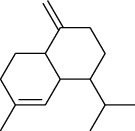	204	–—	–—
58	*α*-Cadinol	C_15_H_26_O	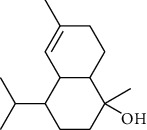	222	–—	–—
59	Bicyclo [3.1.1] hept-2-ene, 3	C_10_H_16_	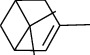	136	–—	–—
60	3-Octenol	C_8_H_14_O	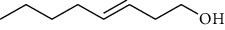	126	–—	–—
61	*α*-Pinene	C_10_H_16_	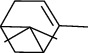	136	–—	–—
62	(+)-Limonene	C_10_H_16_	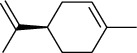	136	–—	–—
63	(E)-*β*-Ocimene	C_10_H_16_	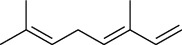	136	–—	–—
64	(R)-3, 7-dimethyl-6-octenol	C_10_H_20_O	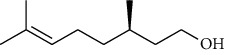	156	–—	–—
65	cis-3, 7-Dimethyl-2, 6-octadienol	C_10_H_18_O	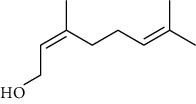	154	–—	–—
66	Bicyclo [3.1.0] hex-3-en-2-ol	C_10_H_16_O	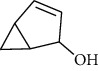	152	–—	–—

**Table 2:** Effects of the TCPT and IPTO of MH on anal temperature change of rats with deficiency of Lung-Yin ($\bar{x}\pm s$, *n* = 10).

Group	Day 1	Day 9	Day 16	Day 25
Model group	36.263 ± 0.478^▲▲^	34.950 ± 0.605^▲▲^	34.738 ± 0.307^▲▲^	34.588 ± 0.189^▲^
TCPT group	35.912 ± 0.196	34.975 ± 0.557	34.575 ± 0.249	34.637 ± 0.602
IPTO group	35.888 ± 0.323	34.925 ± 0.362	34.513 ± 0.391	34.099 ± 0.064 ^*∗*^
Positive group	35.963 ± 0.366	34.675 ± 0.333	34.450 ± 0.355	34.088 ± 0.035 ^*∗*^
Tween-80	36.329 ± 0.519	34.986 ± 0.241	34.571 ± 0.250	34.486 ± 0.524
Normal group	34.780 ± 0.390	34.180 ± 0.915	33.760 ± 0.472	33.980 ± 0.239

^▲^ *P* < 0.05, ^▲▲^*P* < 0.01 vs. normal group, ^*∗*^*P* < 0.05, ^*∗∗*^*P* < 0.01 vs. model group.

**Table 3:** Effects of the IPTO and TCPT of MH on IL-1, IL-1*β*, cAMP, cGMP, and cAMP/cGMP levels of rats with deficiency of Lung-Yin ($\bar{x}\pm s$, *n* = 10).

Group	cAMP/(pmoL·mL^−1^)	cGMP/(nmoL·L^−1^)	IL-1/(pg·mL^−1^)	IL-1*β*/(ng·L^−1^)	cAMP/cGMP(nmoL·L^−1^)
Model group	80.327 ± 6.333^▲▲^	9.054 ± 2.438^▲▲^	59.887 ± 4.940^▲▲^	15.550 ± 1.706^▲▲^	7.854 ± 0.218^▲▲^
TCPT group	77.772 ± 13.382	12.483 ± 1.887 ^*∗∗*^	56.436 ± 11.239^▲^	15.047 ± 2.085^▲^	6.323 ± 0.546 ^*∗*^
IPTO group	75.272 ± 12.251	12.489 ± 0.726 ^*∗∗*^	53.781 ± 4.789	13.952 ± 0.560 ^*∗∗*^	5.980 ± 0.901 ^*∗∗*^
Positive group	68.906 ± 5.603 ^*∗*^	11.558 ± 1.465 ^*∗∗*^	46.289 ± 7.200 ^*∗∗*^	13.126 ± 0.528 ^*∗∗*^	5.835 ± 0.746 ^*∗∗*^
Tween-80	78.786 ± 13.459	9.576 ± 1.695^▲▲^	53.737 ± 15.490	15.403 ± 2.160^▲▲^	8.153 ± 0.926^▲▲^
Normal group	63.924 ± 10.806 ^*∗*^	13.070 ± 0.993 ^*∗∗*^	45.042 ± 7.813 ^*∗∗*^	13.945 ± 0.592 ^*∗∗*^	4.847 ± 0.196 ^*∗∗*^

^▲^ *P* < 0.05, ^▲▲^*P* < 0.01 vs. normal group, ^*∗*^*P* < 0.05, ^*∗∗*^*P* < 0.01 vs. model group.

**Table 4:** Effects of the IPTO and TCPT of MH on CHO, ALB, TP, and BUN levels of rats with deficiency of Lung-Yin ($\bar{x}\pm s$, *n* = 10).

Group	CHO/(mmoL·L^−1^)	ALB/(g·L^−1^)	TP/(g·L^−1^)	BUN/(mmoL·L^−1^)
Model group	2.277 ± 0.139^▲^	31.986 ± 2.315^▲▲^	41.559 ± 2.409^▲▲^	15.949 ± 0.687 ^*∗∗*^^▲^
TCPT group	2.266 ± 0.096^▲^	33.869 ± 2.320^▲▲^	43.087 ± 2.053^▲^	11.628 ± 3.459 ^*∗∗*^
IPTO group	2.016 ± 0.229 ^*∗*^	35.711 ± 0.621 ^*∗∗*^^▲^	45.053 ± 1.694 ^*∗*^	10.965 ± 0.335 ^*∗∗*^^▲^
Positive group	1.935 ± 0.115 ^*∗∗*^	36.430 ± 2.629 ^*∗∗*^	45.099 ± 1.227 ^*∗*^	9.995 ± 1.887 ^*∗∗*^^▲^
Tween-80	2.309 ± 0.363^▲▲^	33.760 ± 2.312^▲▲^	43.895 ± 3.418	14.545 ± 1.167
Normal group	1.926 ± 0.113 ^*∗*^	37.019 ± 1.606 ^*∗∗*^	47.252 ± 2.526 ^*∗∗*^	13.649 ± 1.469 ^*∗∗*^

^▲^ *P* < 0.05, ^▲▲^*P* < 0.01 vs. normal group, ^*∗*^*P* < 0.05, ^*∗∗*^*P* < 0.01 vs. model group.

**Table 5:** The expression of TNF-*α* lung tissue in rats of each groups ($\bar{x}\pm s$, *n* = 10).

Group	Average optical density	Abnormal index
Model group	0.557 ± 0.061^▲^	2.692 ± 0.175^▲▲^
TCPT group	0.474 ± 0.082	2.290 ± 0.209 ^*∗*^
IPTO group	0.460 ± 0.131	2.041 ± 0.229 ^*∗∗*^
Positive group	0.411 ± 0.191 ^*∗*^	2.252 ± 0.231 ^*∗*^
Tween-80 group	0.497 ± 0.035	2.415 ± 0.479
Normal group	0.416 ± 0.070 ^*∗*^	2.130 ± 0.494 ^*∗∗*^

^▲^ *P* < 0.05, ^▲▲^*P* < 0.01 vs. normal group, ^*∗*^*P* < 0.05, ^*∗∗*^*P* < 0.01 vs. model group.

**Table 6:** Comparison of organ coefficients of the heart, spleen, lung, and kidney ($\bar{x}\pm s$, *n* = 10, g/100 g).

Group	Heart	Liver	Spleen	Lung	Renal
Model group	0.351 ± 0.029^▲▲^	3.993 ± 0.498^▲▲^	0.222 ± 0.031^▲^	0.637 ± 0.062^▲▲^	0.736 ± 0.063^▲^
TCPT group	0.327 ± 0.026	3.879 ± 0.390	0.213 ± 0.021	0.601 ± 0.103^▲^	0.719 ± 0.053
IPTO group	0.315 ± 0.020 ^*∗*^	3.709 ± 0.333	0.205 ± 0.019	0.559 ± 0.070 ^*∗*^	0.698 ± 0.046
Positive group	0.315 ± 0.025 ^*∗*^	3.305 ± 0.122 ^*∗∗*^	0.195 ± 0.014 ^*∗*^	0.567 ± 0.043 ^*∗*^	0.677 ± 0.041 ^*∗*^
Tween-80	0.348 ± 0.031^▲▲^	3.874 ± 0.256^▲▲^	0.207 ± 0.020	0.585 ± 0.056^▲^	0.732 ± 0.065^▲^
Normal group	0.294 ± 0.026 ^*∗∗*^	3.269 ± 0.232 ^*∗∗*^	0.191 ± 0.013 ^*∗*^	0.491 ± 0.074 ^*∗∗*^	0.673 ± 0.067 ^*∗*^

^▲^ *P* < 0.05, ^▲▲^*P* < 0.01 vs. normal group, ^*∗*^*P* < 0.05, ^*∗∗*^*P* < 0.01 vs. model group.

## Data Availability

The data used to support the findings of this study are available from the corresponding author upon request.

## Abbreviations

MH: Moslae Herba
IPTO: Integration processing technology of origin
TCTO: Traditional cutting processing technology
GC-MS: Gas chromatography-mass spectrometry
H & E: Hematoxylin and eosin
TNF-*α*: Tumor necrosis factor-*α*
CHO: Cholesterol
ALB: Albumin
TP: Total protein
BUN: Blood urea nitrogen
IL-1: Interleukin-1
IL-1*β*: Interleukin-1*β*
cAMP: Cyclic adenosine monophosphate
cGMP: Cyclic guanosine monophosphate
TC: Serum total cholesterol
TG: Triglyceride
SPF: Sixty specific pathogen-free
SD: Sprague Dawley.
